# Assessing Surgical Care under the Government‑Funded Health Insurance Scheme—Pradhan Mantri Jan Arogya Yojana in India

**DOI:** 10.5334/aogh.4908

**Published:** 2025-12-12

**Authors:** Himanshu Iyer, Uma Gupta, Shreyas Patil, Lokesh Krishna, Sweta Dubey, Siddhesh Zadey

**Affiliations:** 1 Association for Socially Applicable Research (ASAR), Pune, Maharashtra, India; 2 Department of Community Medicine and Family Medicine, All India Institute of Medical Sciences, Bibinagar, Telangana, India; 3 Department of Community Medicine, Seth GS Medical College & KEM Hospital, Mumbai, Maharashtra, India; 4 SUNY Downstate Health Sciences University, Brooklyn, New York, USA; 5 Global Emergency Medicine Innovation and Implementation (GEMINI) Research Center, Duke University, Durham, North Carolina, USA; 6 Department of Epidemiology, Mailman School of Public Health, Columbia University, NYC NY USA; 7 Dr. D. Y. Patil Dental College and Hospital, Dr. D. Y. Patil Vidyapeeth, Pune, Maharashtra, India

**Keywords:** Global surgery, India, Pradhan Mantri Jan Arogya Yojana (PMJAY), surgical planning, low- and middle-income countries, health equity

## Abstract

*Background:* Pradhan Mantri Jan Arogya Yojana (PMJAY) is the world’s largest publicly funded health insurance scheme with over 500 million beneficiaries. It was formulated to provide financial risk protection against health expenditures among the socio‑economically bottom 40% of the Indian population.

*Objective:* To understand equity in surgical care provision under PMJAY by examining patterns across five dimensions: health sector, gender, age, surgical specialty, and geographical distribution.

*Methodology:* We reviewed multiple policy briefs and working papers by the National Health Authority.

*Results:* For both supply and utilization sides, PMJAY is predominantly about surgical care services. About 65% of procedures listed in the scheme cover surgeries and ~82% empaneled hospitals provide surgical care. Regardless of the health sector, over two‑thirds of claims by volume and value were raised for surgical patients. However, key differences and disparities exist in utilization across dimensions. Men have 23.6% greater surgical claims than women beneficiaries, after excluding obstetric and gynecological surgeries. Regional disparities exist with states like Bihar, Madhya Pradesh, and Uttar Pradesh having lower service utilization compared with Gujarat and Kerala. Only 7% of hospitals in “Aspirational Districts” offer specialized surgeries such as cardiothoracic and vascular, compared to 17% in other districts.

*Conclusion:* PMJAY mirrors the broader inequities in Indian healthcare and society. Its role in financing surgical care is significant, yet uneven. To ensure equity and progress toward universal health coverage, proactive steps such as better monitoring and evaluation of disaggregated data, targeted enrollment of individuals in the bottom quintiles of the “bottom 40%,” and increased allocation to surgical services are needed. PMJAY must evolve not just as a financial safety net but as a systemic driver of equitable surgical care.

## Introduction

The lack of affordable healthcare is a significant barrier to achieving universal health coverage (UHC) in several low‑ and middle‑income countries (LMICs) including India. The common indicators of health financing include risks of catastrophic and impoverishing health expenditures (CHE and IHE). CHE is defined as the proportion of the population with out‑of‑pocket expenditure (OOPE) greater than 10% of their pre‑health shock incomes. IHE is defined as the proportion of the population with OOPE greater than the national poverty line (or other relevant international) threshold [1]. With a population of about 1.4 billion, limited investment in public healthcare, and evident socio‑economic inequalities, India contributes to over one‑third of the global population facing CHE and IHE, respectively [2].

Surgical conditions are known to contribute heavily to the financial burden of healthcare seekers, with the average OOPE on surgical conditions being about 1.7 times higher than that on non‑surgical conditions [3]. Previous modeling estimates suggest that 701 million Indians (59.6% of the Indian population) face CHE due to surgical care, while 456 million people (36.5% of Indians) face the risk of IHE due to surgery [1]. Risks for CHE and IHE are also greater for surgery than other healthcare treatments. For instance, a 2014 nationally representative survey found that 24.9% of people seeking healthcare (surgical and non‑surgical treatments) face CHE risk [4]. Another 2011–2012 pan‑India analysis noted that expenditure on medical drugs accounted for 11.2% of CHE [5]. Hence, compared to these estimates, CHE from surgery is about 5.3 times higher than that for medicines and 2.3 times higher than the overall CHE [1, 5]. More critically, the risks for CHE and IHE from surgery are highest for people in the poorest wealth quintile (89.8% and 100%, respectively) [1]. Hence, there is a clear need for financial risk protection directed specifically toward surgical care in India.

The last seven decades have noted limited health policy and planning attention to surgical care in India with the most recent National Health Policy (2017) [6, 7]. The Lancet Commission on Global Surgery (LCoGS) recommended developing national surgical, obstetric, and anesthesia plans (NSOAPs) as vertical initiatives aligned with broader health planning that could help prioritize attention and secure resources for surgical care [8]. Health financing is among the key components of the NSOAP framework.

One mechanism for financial risk protection, in the absence of a vertical plan, is the government‑funded health insurance scheme (GFHIS). In 2018, the Finance Ministry of India introduced the largest national GFHIS named Pradhan Mantri Jan Arogya Yojana (PMJAY) under the Ayushman Bharat program [9]. PMJAY provides coverage up to 500,000 INR (Indian national rupees) annually to each household belonging to the socio‑economically bottom 40% of the total population for expenses for hospitalization at secondary and tertiary level care facilities.

We had previously investigated how GFHIS has evolved in the context of UHC in India and the inequities in the early years of PMJAY implementation [10]. However, PMJAY’s role in expanding the surgical care for the most vulnerable sections of the Indian society, remains unassessed. In this review, we summarize the evidence for surgical care provision under PMJAY. In the first section, we briefly introduce PMJAY as a scheme and summarize the aspects of surgical infrastructure and packages. Next, we assess the differences in surgical care provision and utilization under PMJAY across multiple dimensions of equity, including: health sectors (public and private), surgical specialties, gender and age of care‑seeking beneficiaries, and geographic regions or states. Finally, we highlight the gaps, contextualize PMJAY among the extant surgical financing literature, and scope for improvement that could help universalize surgical care access by providing adequate financial risk protection.

## Methods

As noted above, this work was a part of the larger review that assessed the equity in the supply and utilization of services under PMJAY [10]. Associated methods and the equity framework have been previously described there. Briefly, we acquired data from 18 working papers and 16 policy briefs published on the PMJAY website between September 2018 and November 2022. Information relevant to surgical care was extracted from 8 working papers and 7 policy briefs. Additionally, we acquired information from the annual reports, public dashboards released by the National Health Authority, and health benefits package and procedure lists. Together, these documents provide comprehensive information publicly available on the early years of PMJAY implementation.

The policy briefs and working papers point to several common variables, including the number of hospitals offering surgical care services and types of procedures, representing the health system’s capacity; the number and costs of claims raised across public and private hospitals and specialties, indicating utilization; and the percentage of mortality and readmission rates across public and private sectors and specialties, reflecting the safety and quality of care. We also present a consolidated dataset of 40 variables extracted from the underlying reports used in this review that can be accessed at Harvard Dataverse [11].

## Results

### PMJAY and surgical care

Globally, PMJAY is the largest GFHIS providing coverage up to 500,000 INR (or 6802.7 USD, 1 US dollar or USD = 73.5 INR) annually per household among the 107.4 million eligible households (535 million people or about 40% of the total Indian population). It covers expenses related to secondary‑ and tertiary‑level hospitalizations, including 3 days pre‑hospitalization and 15 days post‑hospitalization. The scheme features paperless and cashless services, portable throughout the country [12]. Eligible PMJAY beneficiaries include: (a) people from rural households belonging to at least 1 of the 6 deprived classes based on the Socio‑Economic and Caste Census (SECC) (https://secc.gov.in/), (b) people from urban households belonging to 1 of the 11 occupational categories under SECC, (c) beneficiaries of the previous GHFIS—Rashtriya Swasthya Bima Yojana (RSBY), and (d) state‑endorsed beneficiaries apart beyond those identified under the SECC criteria [13].

As of 2021, 33 of the (then) 36 states and union territories (UTs), except West Bengal, Odisha, and New Delhi, have implemented PMJAY. Nearly 164 million Ayushman Health e‑cards have been issued to unique eligible beneficiaries. 26,137 hospitals (about 2 hospitals per 100,000 people) have been empaneled (47% private and 53% public), recording 2 million hospital admissions leading to treatments worth 561 billion INR (76 million USD) [14].

**Figure 1:**
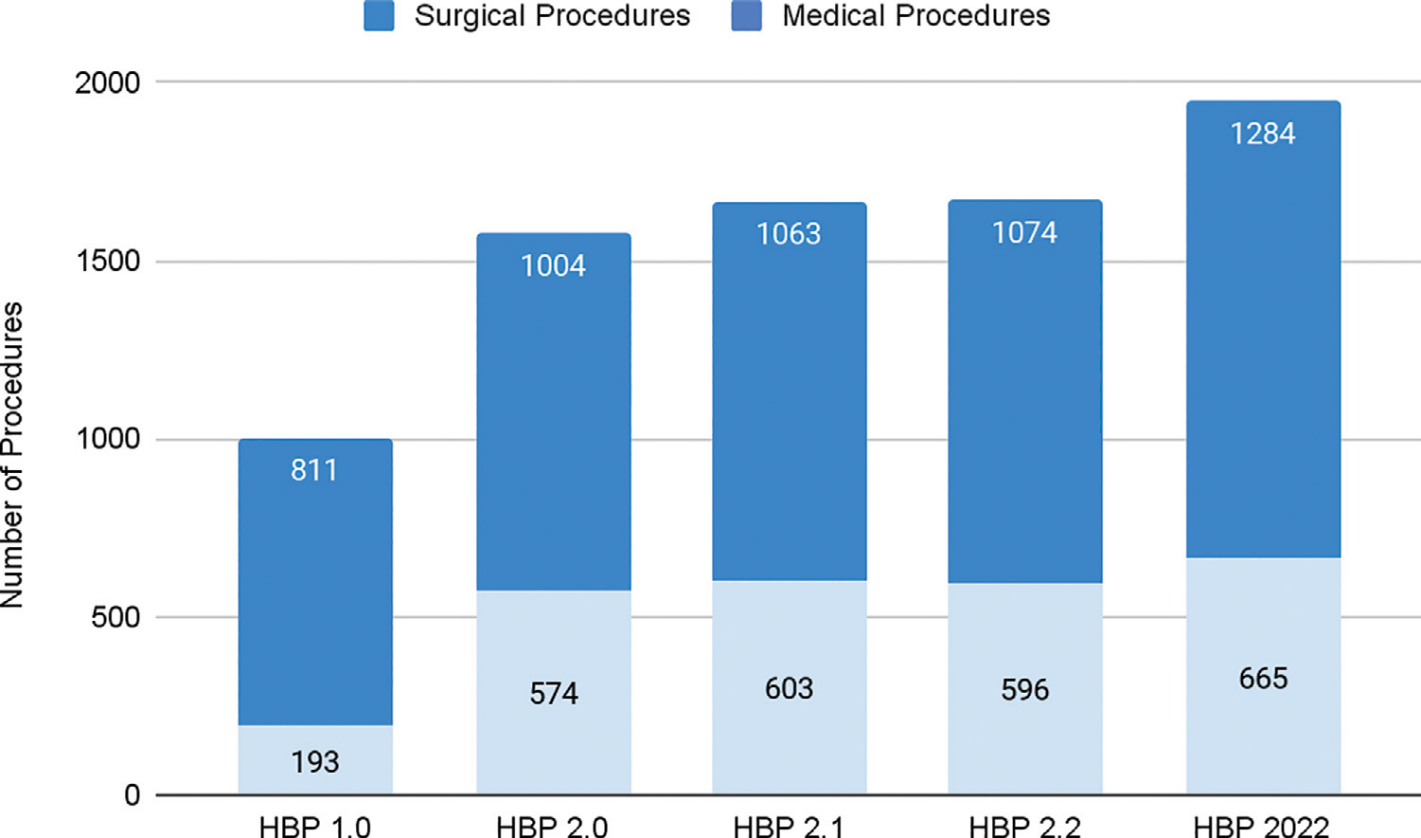
Procedure distribution across different versions of Health Benefits Packages over the years. All surgical and minimally invasive procedures, including cardiology and interventional neurology, were considered under surgical procedures.

The packages and treatments covered by the scheme have changed over the last couple of years with the expansion of package and procedure lists and improved clarity in package descriptions (Figure 1). For uniformity, we considered the procedures enlisted in the health benefits packages (HBPs). The HBP list 1.0 noted 1350 procedures of which about 21.5% were surgical. Both total procedures and the share of surgical specialties increased across HBP iterations. The most recent HBP 2022 listed over 1949 procedures, of which 1284 (65.8%) were surgical and minimally invasive [15]. Surgical interventions not covered in the standard surgical care packages can be booked under an “Unspecified Surgical Package” [12, 13, 15]. Procedures costing less than 100,000 INR (1360 USD) can be directly booked, and those costing more may be approved after thorough scrutiny. The evidence generated from the use of this package was used to incorporate the most booked procedures into the updated HBP. Data from September 23, 2018 to November 30, 2020 on unspecified package utilization noted that it constituted less than 1% of the overall utilization under PMJAY since its launch. There were also notable differences across states based on how early the states participated in the scheme and whether they had existing infrastructure and experience to support implementing a GFHIS. Between September 2018 and November 2020, unspecified package claims were highest in Punjab (41%) followed by Kerala (29%). Chhattisgarh accounted for 7% of claims, and Jharkhand and Assam contributed 3% each [16]. The addition of new packages to HBP 2.0, including the common procedures previously listed under unspecified procedures in HBP 1.0 further reduced the use of unspecified packages across early adopting states predominantly using them [17].

**Figure 2:**
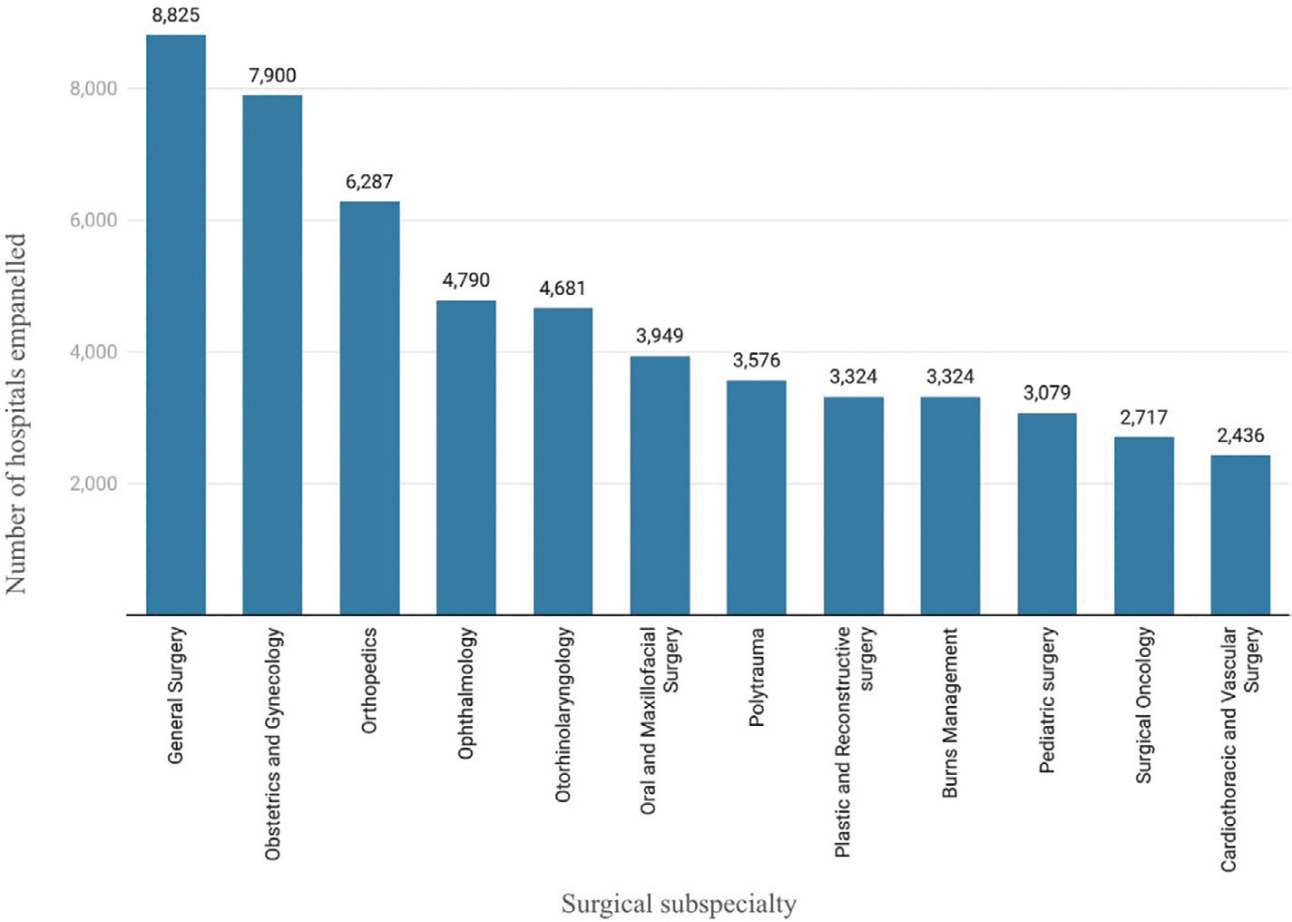
Empaneled hospitals under Pradhan Mantri Jan Arogya Yojana providing surgical services per PMJAY Hospital Dashboard 2021.

The distribution of different surgical specialty packages varies across empaneled hospitals [13]. More hospitals were empaneled for general surgery, obstetrics and gynecology (OBGYN), and ophthalmic care packages than burns management, pediatric surgery, and surgical oncology among others (Figure 2). The costs of surgical and medical packages vary significantly across specialties. However, surgical packages were generally costlier than medical packages. The cheapest surgical (oral and maxillofacial surgery) package covering the extraction of impacted teeth under local anesthesia costs 500 INR (~ 7 USD). While the most expensive packages under cardiothoracic and vascular surgery (CTVS) for the surgical correction of category III congenital heart disease, anomalous left coronary artery from the pulmonary artery by right ventricle–pulmonary artery conduit costs 320,000 INR (4354 USD) [18].

### Surgical care equity across dimensions

#### Private and public surgical care hospitals

**Figure 3:**
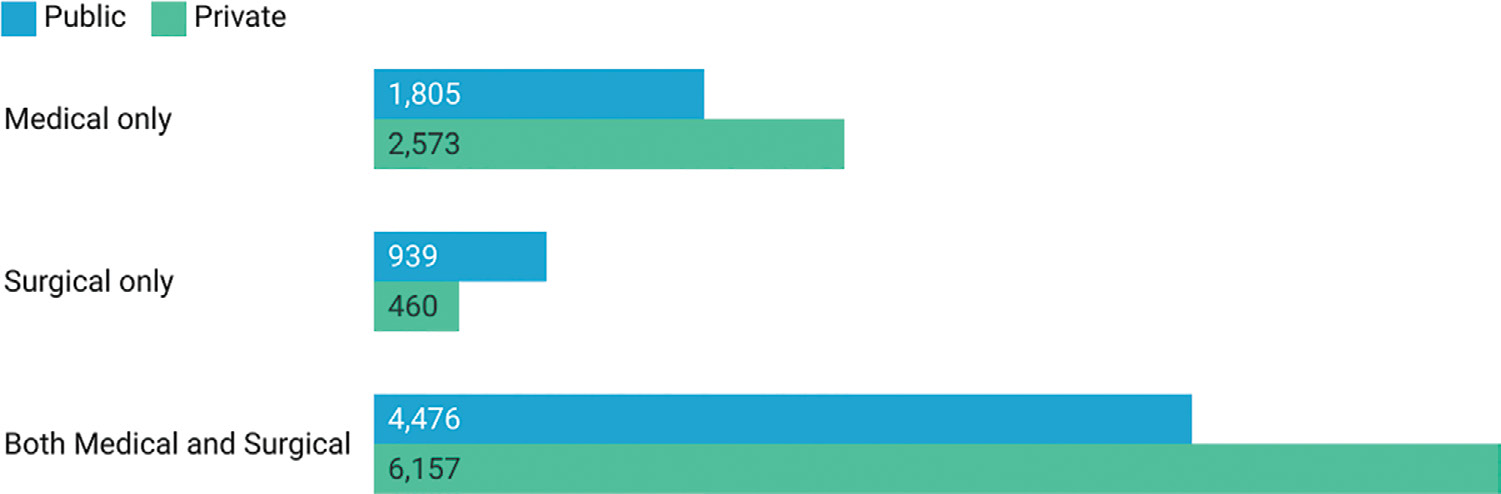
Number of PMJAY empaneled hospitals providing medical and surgical care services from September 23, 2018 to February 29, 2020 [19].

From September 23, 2018 to February 29, 2020, 56% of the 16,410 empaneled hospitals reported were private [19]. Of the 9190 private hospitals, 6157 (67%) provided both medical and surgical care, 2573 (28%) provided only surgical care, and 460 private hospitals (5%) only provided medical care. Of public hospitals, 62% (4476) provided both medical and surgical care, 13% (939) provided only surgical care, and 25% (1805) provided only medical care (Figure 3). Overall, 95% of private empaneled hospitals and 75% of public hospitals provided surgical care services [19].

Private hospitals report several folds of surgical care claim volume and value compared to public hospitals. Greater surgical claim volumes and values at private hospitals may be attributed to their better availability of resources and infrastructure. For instance, 80% of hospitals with an operating room and 75% of hospitals reporting a blood bank were private. This was also evident from the distribution of specialties that the hospitals across two sectors were empaneled to provide. Private hospitals constitute 66% of total hospitals empaneled for CTVS, 82% for neurosurgery, 79% for plastic and reconstructive surgery, and 75% for surgical oncology [19].

Till February 2020, 64% (3,962,845) of claims at private hospitals and 46% (1,683,638) of claims at public hospitals were directed to surgical packages. Surgical packages accounted for 77% of the claim value (82.29 billion INR or 1.12 billion USD) reimbursed to private hospitals and 66% of the claim value (23.76 billion INR or 323.27 million USD) reimbursed to public hospitals [19]. The differences between the claim value across medical and surgical were also noteworthy, where such comparison was possible. Claim values across states for medical oncology ranged from 3000 to 30,147 INR (41–412 USD) at public hospitals and from 5000 to 50,101 INR (68.35–684.90 USD) at private hospitals. Surgical oncology claims ranged from 12,000 to 63,700 INR (163.26–866.66 USD) in public hospitals and from 25,550 to 223,244 INR (347.61–3037.33 USD) in private hospitals. Hence, the high surgical costs were skewed toward private hospitals.

Claims from general surgery (938,616), ophthalmology (472,674), and unspecified packages (392,212) accounted for a sizable portion of surgical claim volume raised by private hospitals. On the contrary, public hospitals had a substantial claim volume share from OBGYN (357,579) [19]. Private hospitals raised a major proportion of claims for cataract procedures (86%), fracture‑hip internal fixation (86%), and percutaneous transluminal coronary angioplasty (PTCA)—single stents (82.5%). While public hospitals dominated the claim volumes for conventional tubectomy (78%) and cesarean deliveries (67%). Between the two common cataract procedures, phacoemulsification (PHACO) and small incision cataract surgery (SICS), 83% of 94,760 PHACO claims were from private hospitals [20]. This may be attributed to the minimally invasive technique, higher cost, and perceived higher effectiveness. It also indicates increased consumer awareness and demand for more technology‑based surgeries. Public hospitals accounted for 77% of the 4,966 requests for SICS cataract surgery, a relatively inexpensive procedure. From September 2018 to May 2019, of the 17,333 hysterectomy claims, 68.7% were from the private sector [21]. Private hospitals also accounted for 73% of claims for cholecystectomy and 58% of claims for appendicectomy [22]. Together, these findings depict that the private sector hospitals empaneled under PMJAY provide more surgeries, including surgeries that were more complex and expensive.

**Figure 4:**
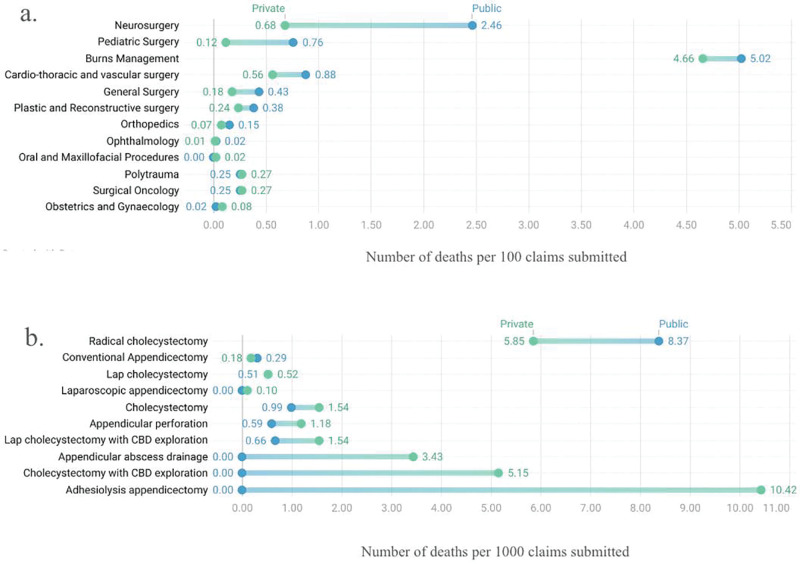
Mortality proportions (%) in public and private hospitals for different **(a)** surgical subspecialties and **(b)** surgical procedures [22, 23].

Beyond claim volume and value, public and private hospitals also differ in surgical quality indicators. The average length of stay in private hospitals for surgical procedures (7.1 days) was shorter than in public hospitals (8.7 days) [19]. From September 23, 2018 to November 30, 2019, in‑hospital mortality rates in public and private hospitals were 0.7% and 0.5%, respectively. Mortality rates were higher in public hospitals for burns management, CTVS, general surgery, neurosurgery, ophthalmology, orthopedics, pediatric surgery, plastic and reconstructive surgery, and surgical oncology. Conversely, mortality rates in public hospitals were lower compared to their private counterparts for OBGYN, oral and maxillofacial surgery, and polytrauma (Figure 4a) [23]. Procedure‑specific mortality rates also showed higher mortality rates in public hospitals than private for: adhesiolysis appendicectomy, appendicular abscess drainage, appendicular perforation, laparoscopic appendicectomy, cholecystectomy, cholecystectomy with common bile duct exploration, and laparoscopic cholecystectomy with common bile duct exploration. Mortality rates were lower in public hospitals for radical cholecystectomy (Figure 4b) [22]. The wide differences in mortality in private–public hospitals for neurosurgery, pediatric surgery, burns management, and CTVS may be associated with the quality of surgical and post‑surgical care [23].

Readmission rates for claims through October 31, 2019, noted that medical procedures across both private and public hospitals had a higher 30‑day readmission rate (8.4%) than surgical procedures (3.2%). The 30‑day readmission rates were marginally higher in private hospitals (5.2%) compared to public hospitals (5%). Surgical and post‑operative complications were the predominant factors driving readmissions for surgical cases [23].

#### Surgical subspecialties

PMJAY Transaction Management System revealed that from September 23, 2018 to May 15, 2019, surgical packages accounted for 61% and 75% of all claims by volume and monetary value, respectively [24]. Among high‑value claims, i.e., claims with a value > 30,000 INR (408 USD), 79% were for surgical packages (by volume and value). For claims valued at > 100,000 INR (1360 USD), this proportion increased to 83% for both claim volume and value [24].

Out of 5 of the high‑value specialties (Cardiology, CTVS, Orthopedics, and Neurosurgery) and very high‑value specialties (Cardiology, CTVS, Orthopedics, and Interventional Neurology), 4 were surgical. Out of the 10 high‑value packages (Coronary Balloon Angioplasty, PTCA with the stent, PTCA—single stent, total knee replacement, percutaneous nephrolithotomy, and PTCA—double stent), 6 were surgical, and 7 out of 10 very high‑value claims packages (coronary artery bypass graft (CABG) on pump, PTCA—double stent, mitral valve replacement with valve, mitral valve replacement with a mechanical valve, primary knee replacement with an implant, and CABG off‑pump) were surgical. Hence, PMJAY utilization in terms of volumes and monetary value has been predominantly surgical [24]. General surgery, CTVS, surgical oncology, and orthopedics had relatively greater procedure shares. General surgery and OBGYN packages that can be provided by secondary‑level and above hospitals were more common than those listed under CTVS, pediatric surgery, and surgical oncology.

Certain subspecialties have received greater interest across brief reports and working papers on PMJAY. HBP 2.1 covered 163 cardiology and CTVS procedures, which was raised to 182 in the most recent HBP 2022. About 480,000 cardiac care claims accounted for 26% of the total financial expenditure of the scheme. The top five cardiac care packages in terms of claim volume and value were surgical: percutaneous transluminal coronary angioplasty‑single stent (34% by claim volume and 32% by claim value), PTCA‑double J stent (20% and 24%), CABG (9% and 13%), management of acute myocardial infarction (4% and 2%), and mitral valve replacement (3% and 5%) [25].

Of 1004 surgical care procedures offered under HBP 2.0, 230 (22.9%) were under surgical oncology—an important surgical subspecialty. Surgical oncology procedures contributed 43.4% to the total oncology procedures noting the importance of surgery within oncological services. However, for conditions with cancer that have complex treatment management protocols, a greater proportion of procedures on the supply side cannot be taken as a proxy for need and utilization. For example, from September 2018 to July 2019, surgical oncology accounted for 3.3% of the total 1,88,409 oncology claims [26].

As noted above, the overall surgical readmission rate was 3.2%. Among surgical subspecialties, interventional neuroradiology (11.4%) had the highest readmission rate followed by plastic and reconstructive surgery (7.3%) and urology (7.1%), whereas OBGYN had the lowest readmission rate (1.1%) [23]. The 30‑day readmission rates for the top three procedures contributing most the readmissions were 23.7% for ureteroscopy with stone removal by lithotripsy, 11.2% for double J stent unilateral, including cystoscopy, ureteric catheterization and retrograde pyelogram, and 2.5% for PTCA with single stent [23].

#### Gender of surgical care seekers

The previously existing social and government‑funded health insurance schemes in India, such as the Employee State Insurance Scheme (ESIS) and the Central Government Health Scheme (CGHS) have exhibited a bias in utilization favoring working‑class men over women [27]. However, PMJAY has had early success in achieving gender‑equitable utilization. Scheme design has a role to play since eligibility criteria include households without male members.

Between September 2018 and December 2019, data from 31 states and union territories (excluding Rajasthan and Andhra Pradesh, where gender‑based data was unavailable) revealed some interesting trends. From September 2018 to December 2019, out of the 51,91,239 preauthorizations raised, 25,17,899 claims (48.5%) were for women and 26,73,251 claims (51.5%) were for men [27]. Of the total claims paid at 43,528 million INR (592 million USD), 24,213 million INR (329 million USD) or 56% was directed towards men, while 19,315 million INR (263 million USD) or 44% was for women. This indicates that a 3% point difference in the claim volumes across men and women has resulted in an 11.2% difference in claim value [27]. There were notable differences in surgical services availed by men and women mainly due to the utilization of OBGYN services among women. In the given period, 35.5% of surgical claims raised by women were for OBGYN surgeries while surgical claims contributed to 55.7% of all (surgical, medical, and unspecified) claims by women. When OBGYN surgeries were excluded, men accounted for 14,61,239 (61.8%) surgical procedures, while women accounted for 904,846 (38.2%), indicating a 23.6% difference [27].

**Figure 5:**
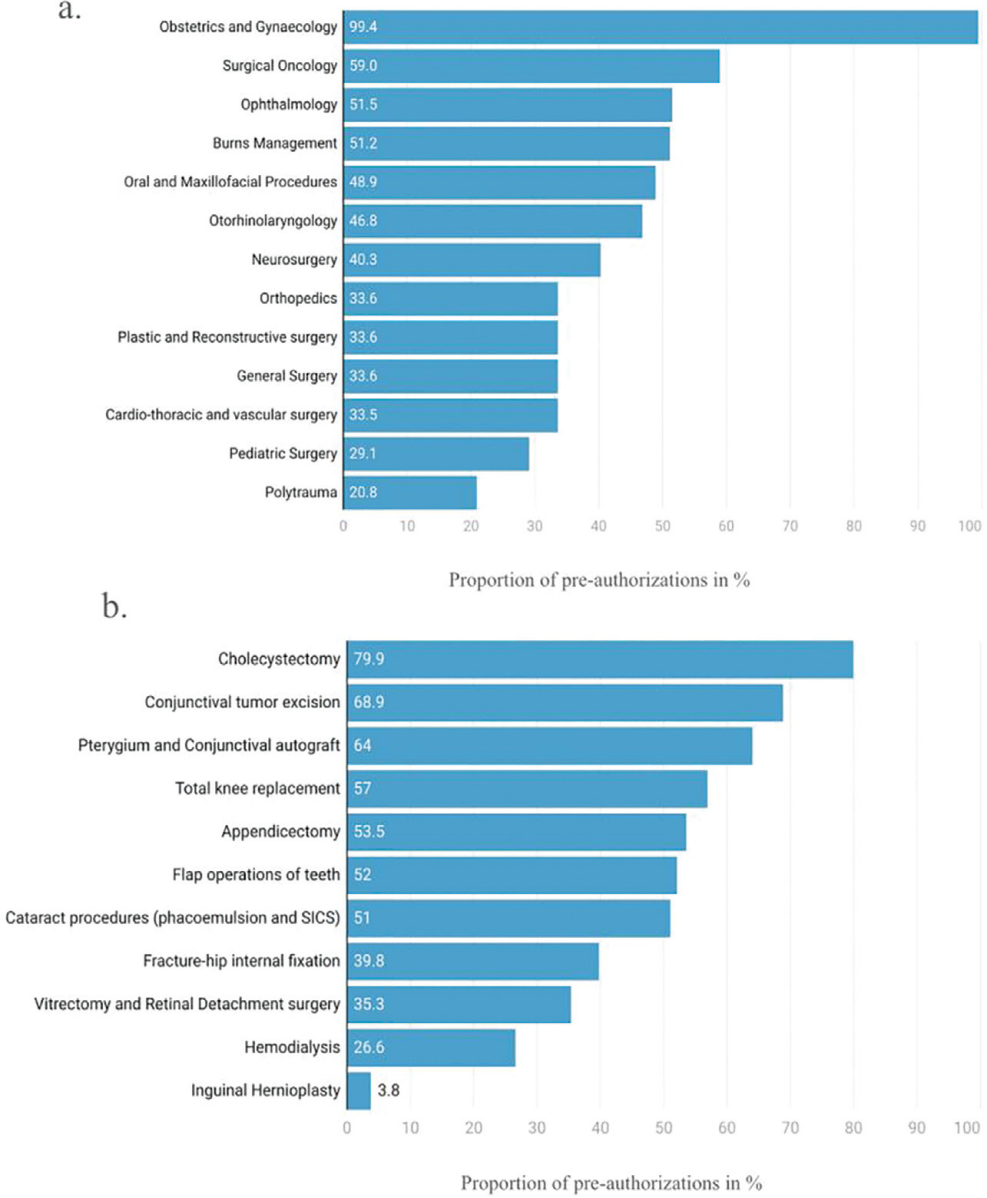
Claims distribution (%) raised under PMJAY by women across **(a)** surgical subspecialties and **(b)** surgical procedures [27].

A higher proportion of claims for women were observed in subspecialties, including obstetric and gynecologic (OBGYN) surgery, surgical oncology, burns management, and ophthalmology. Conversely, men accounted for a majority of claims in oral and maxillofacial surgery, otorhinolaryngology, neurosurgery, orthopedics, plastic and reconstructive surgery, general surgery, CTVS, and polytrauma [27]. Gender disparities present in the larger society were also reflected in PMJAY utilization with only 29.1% of claims raised for pediatric surgery being for girl children (Figure 5a).

For surgical procedures, high‑value claims among women included cholecystectomy (laparoscopic or others), [22, 27], appendicectomy, cataract removal via the SICS technique, cataract removal via phacoemulsion technique, total knee replacement, and flap operation of teeth (Figure 5b) [27]. In contrast, men accounted for a majority of claims for dialysis, inguinal hernioplasty, and fracture‑hip internal fixation [27]. In total, women accounted for 38% of high‑value claims and 48% of all PMJAY claims [24]. This divergence, with a greater share of high‑value claims attributed to men (62%), may be due to the higher prevalence of cardiovascular diseases among men, leading to increased utilization of cardiac surgery packages that were typically of high‑value (women: 30% vs. men: 70%) [24, 25].

In several states such as Arunachal Pradesh, Meghalaya, Karnataka, Chhattisgarh, Andhra Pradesh, Sikkim, and Mizoram, women utilized overall surgical services more than men. However, this trend reversed for most states after excluding OBGYN. In the given period, 83% of surgical claims raised by women in Bihar, 67% in Meghalaya, and 66% in Mizoram were for OBGYN surgeries, while OBGYN claims contributed to 35.5% of all surgical claims by women across India [27]. Additionally, women made up a larger share of admissions in Aspirational Districts, potentially due to the availability of OBGYN services at the secondary level hospitals. OBGYN was most utilized for non‑portable cases (within the state), suggesting that women were less likely to travel to other states for specialized care [28].

#### Age of surgical care seekers

Surgical needs vary across age groups owing to differences in underlying disease burdens. For instance, congenital diseases requiring surgical intervention were more common among children under 10 years of age, while OBGYN surgeries were more common in women of the reproductive age group (15–49 years). Essential or emergency ophthalmology (such as cataract and glaucoma) and cardiovascular surgery were more common in middle‑aged and elderly individuals. The changing demographics along with the increased burden of non‑communicable diseases in the country will increase the surgical need for both these specialties in the coming decades [23, 29].

The highest utilization of cataract services between January and May 2019 was seen in the 65–85‑year‑old age group (49.04%) [29]. Oncology claims were most common in the age group 45–50 years and 50–55 years for women and men respectively between September 2018 and July 2019 [26]. Similarly, the proportion of claim volume for CTVS packages was highest in the 51–60 age group (30.6%) from September 2018 to March 2020 [25]. The 30–44‑year‑old age group accounted for the highest utilization of surgical packages for stroke management (6.4%) from August 2019 to March 2021, followed by 45–59 years (5.6%) [30]. High‑value packages, which were mostly surgical, were claimed by beneficiaries in the 19–50 years age group (39%) based on the data from September 2018 to May 2019 [24]. The highest utilization of appendectomy packages was in the age group 0–19 years, while cholecystectomy package utilization was maximum in the 30–39‑year‑old women and 40–49‑year‑old men [22]. Nearly 75% of cardiac care (cardiology and CTVS) claim volume was contributed by those aged between 41 and 70 years [25].

Age‑based patterns of utilization were also seen in specialties relevant to women, including breast cancer surgery and hysterectomy. During 2019–2020, the maximum utilization of breast cancer services under PMJAY was by women in the 40–50 years age group [31]. From September 2018 to April 2019, the proportion of hysterectomy claims was highest in the women of age group 40–49 years (46.2%) followed by 30–39 years (24%) and 50–59 years (17.5%) [21]. The proportion of portability claims (i.e., claims by beneficiaries crossing state boundaries) that were more common for subspecialty and surgical services, was greatest for those in the 25–44 years age group and limited for the elderly ( > 65 years) [28].

#### Surgical care across geographic regions

The geographic differences for supply and utilization side characteristics for PMJAY more broadly have been noted elsewhere [10]. Health system, governance, and broader societal factors influence these differences, whose analysis is beyond the scope of the current article. Here, we note some specific instances of such differences in surgical care.

Generally, states with higher poverty headcount ratios and high case load among beneficiaries, such as Bihar, Madhya Pradesh, and Uttar Pradesh, showed limited utilization of medical and surgical services under PMJAY, while the states with lower poverty headcount ratios and disease burden, including Gujarat and Kerala, showed greater utilization. These differences can be partly attributed to differences in health infrastructure [32].

**Figure 6:**
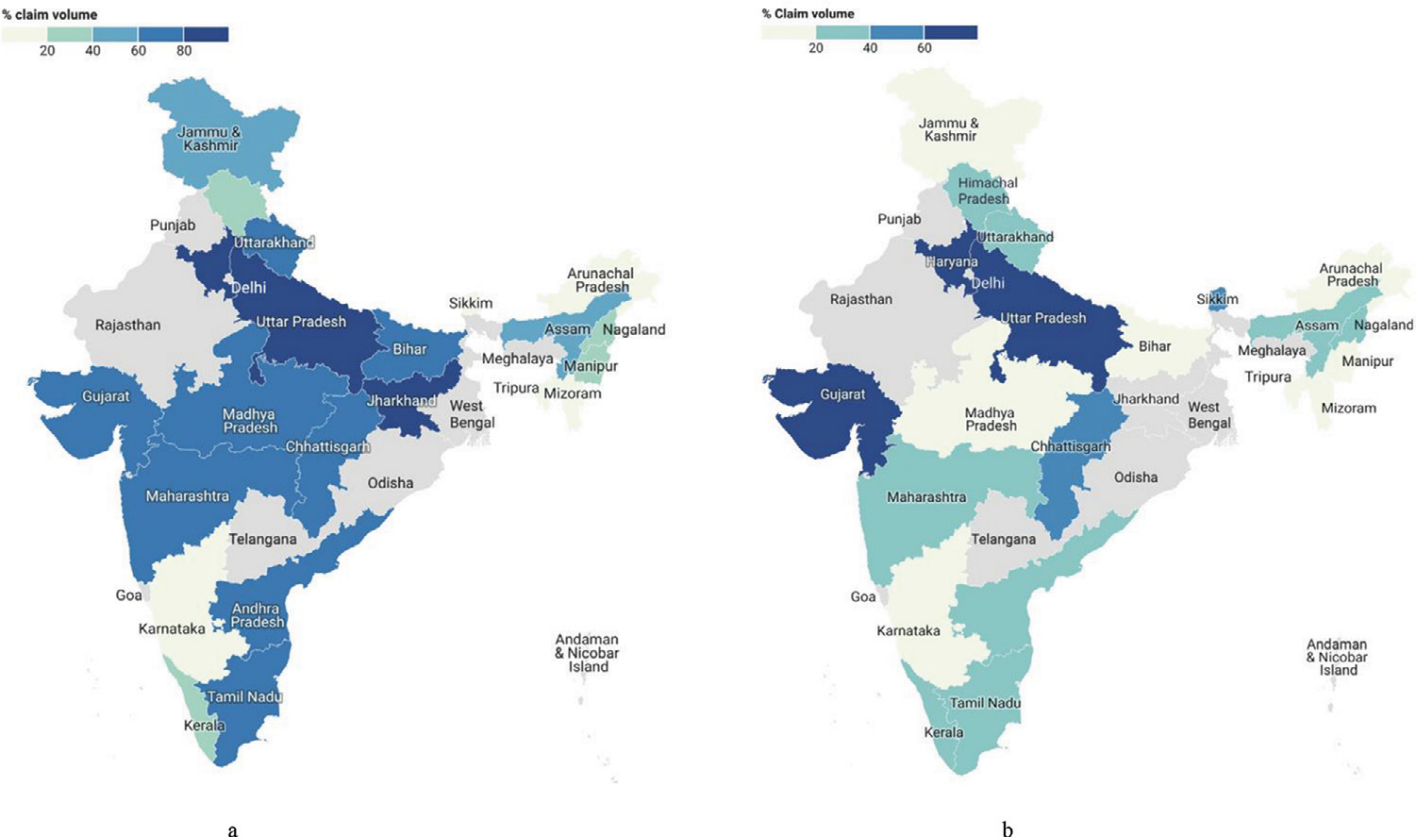
Percentage claim volume at private hospitals across various states under PMJAY for **(a)** general surgery and **(b)** obstetrics and gynecology [19].

The picture was more complicated for different surgical subspecialties and procedures based on local needs, penetration of different sectors, and availability of services. The northeastern states that had only a few private hospitals raised some of the lowest claim volumes (20%) for general surgery services (Figure 6a) [19]. The proportion of claims for general surgery varies from null in Arunachal Pradesh and Tripura to 98% in Jharkhand. Some of the highest claim volume shares for general surgery in private hospitals were in Jharkhand (88%), Gujarat (82%), and Haryana (80%) [19]. The ratios of total surgical claims to surgical claims excluding OBGYN were high in Bihar (2.96), Meghalaya (2.13), and Arunachal Pradesh (1.49) indicating that surgical procedures under OBGYN constituted a major part compared to other surgical subspecialties. Contrastingly, Gujarat (1.05), Maharashtra (1.01), and Tamil Nadu (1.23) observed similar volumes for total surgical and surgical excluding OBGYN claims (Figure 6b) [27].

Hysterectomy is among the common surgeries needed by women. From September 2018 and April 2019, Chhattisgarh accounted for 21.2% of all hysterectomy claims, followed by Uttar Pradesh (18.9%) and Jharkhand (12.2%) [21]. Majority of hysterectomy claims in Chhattisgarh (94.5%) and Jharkhand (96.7%) were made by private hospitals. This contrasts with northeastern states where 100% of claims in Arunachal Pradesh, Meghalaya, Mizoram, and Tripura were made by public hospitals [21]. From January to May 2019, Chhattisgarh, an early PMJAY‑adopting state, accounted for 48.6% of cataract claims, followed by Jharkhand (25.4%), and Uttar Pradesh (11.6%) [29]. Private hospitals accounted for almost all cataract surgery claims in Chhattisgarh (99%), Jharkhand (99%) and Uttar Pradesh (93%). Similar to hysterectomy, for cataract surgeries too, all claims in Chandigarh, Nagaland, Sikkim, and Tripura were from public hospitals. There were only a few private eye care hospitals that existed in the northeastern states [29]. While such descriptive data cannot reveal the exact mechanism, they point to the private sector filling in as the main service provider in states lacking public sector resources, where people eligible to enroll in PMJAY need essential surgical care.

Surgical oncology claims accounted for a majority of oncology claims raised from September 2018 to July 2019 in Karnataka (39.2%), Dadra and Nagar Haveli (19.9%), and Sikkim (18.2%) [26]. This pattern defies the distribution of hospitals providing surgical oncology services under PMJAY, primarily concentrated in Tamil Nadu (42.5%), Maharashtra (17.1%), and Chhattisgarh (7%) [26]. This discrepancy may be attributed to migration to other states for cancer care, as observed in Madhya Pradesh, Uttar Pradesh, Bihar, Jharkhand, Maharashtra, and Dadra and Nagar Haveli. Public hospitals were the primary providers of surgical oncology services in Arunachal Pradesh (33%), Chhattisgarh (19%), Karnataka (43%), and Sikkim (50%). In contrast, Dadra and Nagar Haveli, Daman and Diu, and Goa lacked public hospitals offering such care. Private hospitals accounted for a higher proportion of surgical oncology claims in Manipur (100%), Dadra and Nagar Haveli (21%), and Karnataka (31.5%) [26].

Similar to the states, there were differences across districts. The districts that need a greater push for socio‑economic development, known as Aspirational Districts, have noted limited PMJAY utilization. The low utilization can be attributed to fewer number of hospitals with specialized surgical services in the Aspirational Districts compared to other districts [33]. For instance, from September 2018 to August 2019, 7% of hospitals were empaneled for CTVS and 8% for surgical oncology in Aspirational Districts compared to 17% and 18% in other districts [33]. Such differences make it pertinent that beneficiaries were restricted to their home state or district for availing services.

Portability, a key feature of PMJAY, lets patients seek care at any empaneled hospital across the country regardless of their place of origin or enrollment. Typically, when tertiary‑care hospitals in a beneficiary’s home state opt to remain out of the scheme, beneficiaries can travel to other states to seek the required care. The states that the beneficiaries travel from are called outgoing states, while the states where they receive care are incoming. Out of the top 10 portability specialties (excluding multiple and unspecified packages), 6 were surgical, i.e., Cardiology, CTVS, Orthopedics, Neurosurgery, General Surgery, and Urology. Out of the top 10 portability packages, 7 were surgical [28].

Nationally, 68,669 portability cases (0.97% of total PMJAY cases) with a claim value of 1350 million INR (18.36 million USD) were recorded from September 2018 to December 2019 [28]. The interstate portability proportions for high‑ and very high‑value claims were 2.4% and 5.3%, respectively. 24 Madhya Pradesh and Uttar Pradesh contributed nearly half of the outgoing portable cases by volume and value [28]. Gujarat catered to 26% of incoming volumes that represented 32% of claim value, followed by Uttar Pradesh (9% of volume and 6% of value), Maharashtra (8% for both), and Uttarakhand (7% and 4%) [28]. Madhya Pradesh, Uttar Pradesh, Bihar, Jharkhand, and Haryana account for 80% of the patient movement for cancer care. Bijnor in Uttar Pradesh, the union territory of Dadra & Nagar Haveli, Saharanpur in Uttar Pradesh, Ratlam in Madhya Pradesh, and Garhwa in Jharkhand had the highest number of outgoing cases. Ahmedabad and Vadodara in Gujarat, Dehradun in Uttarakhand, Varanasi in Uttar Pradesh, and Nagpur in Maharashtra were the top districts providing treatment to incoming patients [28]. From September 2018 to December 2019, patients from Uttar Pradesh, Madhya Pradesh, Jharkhand, Haryana, and Bihar moved out of their states to seek surgical oncology services in Gujarat, Maharashtra, and Assam [26].

Higher portability was also noted for subspecialties that were known to have more high‑ and very high‑value packages and procedures. For example, a 2.2% portability rate for cardiac claims indicates that a higher percentage of people need to travel to access quality cardiac care compared to other treatments with the majority of claims from September 2018 to March 2020 raised from Gujarat (29.8%), Maharashtra (22.8%), and Tamil Nadu (13%) [25].

## Discussion

### Main takeaways

Pradhan Mantri Jan Arogya Yojana (PMJAY) has been crucial for improving access to surgical care by scaling up delivery through incentivizing hospitals to build surgical care capacity and ensuring utilization through financial risk protection of surgical care seekers in socio‑economically disadvantaged groups in India. Even so, ensuring equity in PMJAY implementation is important for the benefit of both providers and beneficiaries.

On the delivery side, compared to the public sector, private sector hospitals covered more surgical packages, had a greater share of claims by volume and value, and were favored for specialized surgeries such as neurosurgery and cardiothoracic surgery, reflecting their advanced infrastructure [19]. There are multiple interpretations of these data with differing policy implications.

On one end of the spectrum, an interpretation could be that the private sector is helping expand the overall population health coverage at the population level by opening doors to PMJAY beneficiaries who otherwise could not have afforded the care. The private hospitals might also be easing the burden on the public sector by offering services that might be otherwise unavailable at public facilities, especially for certain specialties. Such interpretations imply that the private sector as the surgical care provider and the government as the payer on behalf of the people could help improve surgical care access. On the other end of the spectrum, the greater role of the private sector in surgical care delivery could also be viewed as detrimental to overall population health, as it could weaken the public health system by competing for financial investments, steer the decision‑making around benchmarking prices toward profits, or weaken the commitment to UHC through other ways. There is growing literature on how privatization leads to worsening of health systems and poor health outcomes [34]. We do not endorse a particular interpretation. Rather, we suggest that regardless of one’s choice of interpretation, these data unequivocally show the important role of the private sector in surgical care delivery. Against this background, the state and national governments can decide whether they want to be payers of health or providers of health depending on their contingencies. However, the government’s regulatory role for PMJAY implementation is vital to ensure health equity and UHC.

Above, we also described in detail the patterns of differences in PMJAY utilization based on gender, age, and place of residence. Some of these differences could be explained as a consequence of underlying differences in the disease burden or needs. For instance, the concentration of cataract surgeries in the older age groups and differences in procedure rates between men and women before excluding OBGYN procedures among others reflect differences. However, there are several instances where the differences are unjust and therefore should be recorded as disparities requiring attention and intervention by the implementation authorities. For instance, the gender‑based differences in the pediatric surgery claims or differences between utilization rates between men and women after excluding OBGYN procedures, and the differences in utilization rates between Aspirational vs. other districts, differences in portability patterns across age groups and across states, and differences in in‑hospital mortality rates between private and public sectors reflect disparities. These disparities, in all likelihood, are present in broader Indian healthcare and society and have made their way into PMJAY. While PMJAY is known to have reduced pro‑rich inequalities in health insurance coverage in rural areas [35], our findings demonstrate that proactive efforts are needed in design adaptations and scheme implementation to mitigate disparities in supply, utilization, and quality of services—especially surgical care services.

### Recommendations

Proactive efforts to reduce disparities in supply, utilization, quality, and other aspects of surgical care services would require routine monitoring and evaluation of disaggregated data on outcomes at appropriate (such as districts) geographic levels. Lack of data by wealth quintiles, caste and religious groups, rural and urban residence, etc. complicates the evaluation of PMJAY’s performance across different socio‑economic and demographic groups. These data deficiencies restrict a comprehensive understanding of the program’s equitable impact. In the current review, we consolidated data across several working papers and policy briefs. More effective assessments would require data on programs, processes, and health outcomes at the local level to identify underserved areas and populations.

There are several decision‑making implications of monitoring and evaluation. For instance, portability is an important feature noting flexibility in access to care under the scheme’s design. However, we noted above that the skew favored men, younger people, and those close and those closer to the smaller urban centers. Tracking portability could also be useful for reconfiguring resource allocation in a needs‑based way. Facilities in incoming states could be assisted with efficient procurement of consumable resources and prioritized for faster claim reimbursements. The outgoing states and districts on the other hand should be prioritized for investments in healthcare infrastructure to ensure equitable supply in the medium‑run. Other advantages of the unified information and communication technology systems and their role in promoting equity have been recently described elsewhere in PMJAY’s context [36].

Better targeted approaches to enroll beneficiaries in the lower quintiles of the bottom 40% are needed to ensure equity in utilization. Such efforts can be informed by several local awareness surveys of potentially eligible beneficiaries that have come up in the last couple of years [37, 38]. On the supply side, the assessments of the readiness of the healthcare facilities and the willingness of healthcare workers are also vital for ensuring grassroots implementation [39].

### PMJAY and global surgery

It is important to contextualize PMJAY in the ongoing discourse on surgical care financing in Global Surgery. Financing is a critical domain in NSOAP and CHE and IHE are considered the financial impact indicators by the LCoGS. More recently, there has been a focus on innovative financing mechanisms, such as public–private partnerships and the introduction of health cess in different contexts with otherwise constrained healthcare spending [40].

In India’s context, PMJAY depicts innovative financing in some aspects. PMJAY operates with a 60:40 spending split between the central and the state governments (except northeastern states, Jammu and Kashmir, Himachal Pradesh, and Uttarakhand with a 90:10 split) [41]. The governments procure health services for the public (eligible beneficiaries) by empaneling public and private sector hospitals. Hence, people can seek surgical care where they want, which is paid for upfront. Further, aligned with the LCoGS recommendations, PMJAY also enhances infrastructure, service delivery, and workforce through improved health financing. For instance, PMJAY generated an annual financial gain of 11.9 million INR (169,607 USD) per district hospital making them self‑sustaining in terms of revenue generation [42]. By directing resources to the publicly owned district hospitals, PMJAY can strengthen health systems and address geographic disparities in access to care. As a national health financing strategy, PMJAY aims to achieve a net financial benefit for both, hospitals and eligible households [42].

Beyond the financing mechanisms and their impacts, at the aggregate level, PMJAY seems to be taking up the role of surgical care financing similar to that taken up by the NSOAP in other countries. In the financial year 2021–2022, India allocated 64,000 crore INR (~ 871 million USD) or about 985 INR (~ 13.3 USD) per capita, representing 8.65% of its total health budget to PMJAY. This allocation is comparable to countries implementing NSOAP, including Rwanda (69.7 million USD per year, i.e., ~ 5.17 USD per capita), Zambia (171.44 million USD per year, i.e., ~ 8.8 USD per capita), and Tanzania (597 million USD per year, i.e., ~ 9.38 USD per capita) [43]. However, Nigeria, which is relatively closer to India for the standard of living and demographic changes, allocates 16.8 billion USD per year, or ~ 78.6 USD per capita, for its NSOAP, i.e., six times greater than India’s allocation to PMJAY. Hence, there is a need to increase overall budget allocation to PMJAY, which has also been previously argued elsewhere [10]. While PMJAY must improve population and cost coverage for achieving UHC, it has made significant strides in India’s efforts to finance surgery.

## Conclusion

To achieve true UHC and address disparities in supply, utilization, and quality of surgical services, PMJAY must evolve beyond a financial safety net and become a catalyst for systemic change. An alternative approach could involve offering essential surgical procedures to the entire population, ensuring basic access for all.

## Data Availability

All data are presented in the manuscript.
